# Electroencephalographic (EEG) Biomarkers in Genetic Neurodevelopmental Disorders

**DOI:** 10.1177/08830738231177386

**Published:** 2023-06-01

**Authors:** Kimberly Goodspeed, Dallas Armstrong, Alison Dolce, Patricia Evans, Rana Said, Peter Tsai, Deepa Sirsi

**Affiliations:** 1 Department of Pediatrics, Division of Neurology, 12334 University of Texas Southwestern Medical Center, Dallas, TX, USA; 2 Department of Neurology, 12334 University of Texas Southwestern Medical Center, Dallas, TX, USA; 3 Department of Psychiatry, 12334 University of Texas Southwestern Medical Center, Dallas, TX, USA; 4 Department of Neuroscience, University of Texas Southwestern Medical Center, Dallas, TX, USA

## Abstract

Collectively, neurodevelopmental disorders are highly prevalent, but more than a third of neurodevelopmental disorders have an identifiable genetic etiology, each of which is individually rare. The genes associated with neurodevelopmental disorders are often involved in early brain development, neuronal signaling, or synaptic plasticity. Novel treatments for many genetic neurodevelopmental disorders are being developed, but disease-relevant clinical outcome assessments and biomarkers are limited. Electroencephalography (EEG) is a promising noninvasive potential biomarker of brain function. It has been used extensively in epileptic disorders, but its application in neurodevelopmental disorders needs further investigation. In this review, we explore the use of EEG in 3 of the most prevalent genetic neurodevelopmental disorders—Angelman syndrome, Rett syndrome, and fragile X syndrome. Quantitative analyses of EEGs, such as power spectral analysis or measures of connectivity, can quantify EEG signatures seen on qualitative review and potentially correlate with phenotypes. In both Angelman syndrome and Rett syndrome, increased delta power on spectral analysis has correlated with clinical markers of disease severity including developmental disability and seizure burden, whereas spectral power analysis on EEG in fragile X syndrome tends to demonstrate abnormalities in gamma power. Further studies are needed to establish reliable relationships between quantitative EEG biomarkers and clinical phenotypes in rare genetic neurodevelopmental disorders.

## Introduction

One in 6 children in the United States have a developmental disability.^1^ Developmental delay is defined as a delay in acquisition of language, social, or motor skills and may present with or without cognitive impairments. More than one-third of children with a neurodevelopmental disorder are identified to have a causative genetic etiology, most of which disrupt early brain development, neuronal signaling, or synaptic plasticity.^2–4^ To date, care remains supportive and is focused on symptomatic management of behavior, sleep, and developmental disruption with speech, occupational, physical, or behavioral therapy as the mainstays of treatment. Development of precision or targeted therapies for rare genetic neurodevelopmental disorders are rapidly being developed.^5–8^ However, clinical trials focused on neurodevelopmental disorders face many challenges including phenotypic heterogeneity, small effect size in clinical outcome measures, lack of shared translatable outcome measures between animal models and human patients, timing of intervention, duration of the clinical trial to detect delayed benefits, and selection of appropriate patients.^9^ Electroencephalography (EEG) is a promising noninvasive surrogate biomarker of brain dysfunction in neurodevelopmental and neuropsychiatric disorders.^10–12^

**Figure 1.**
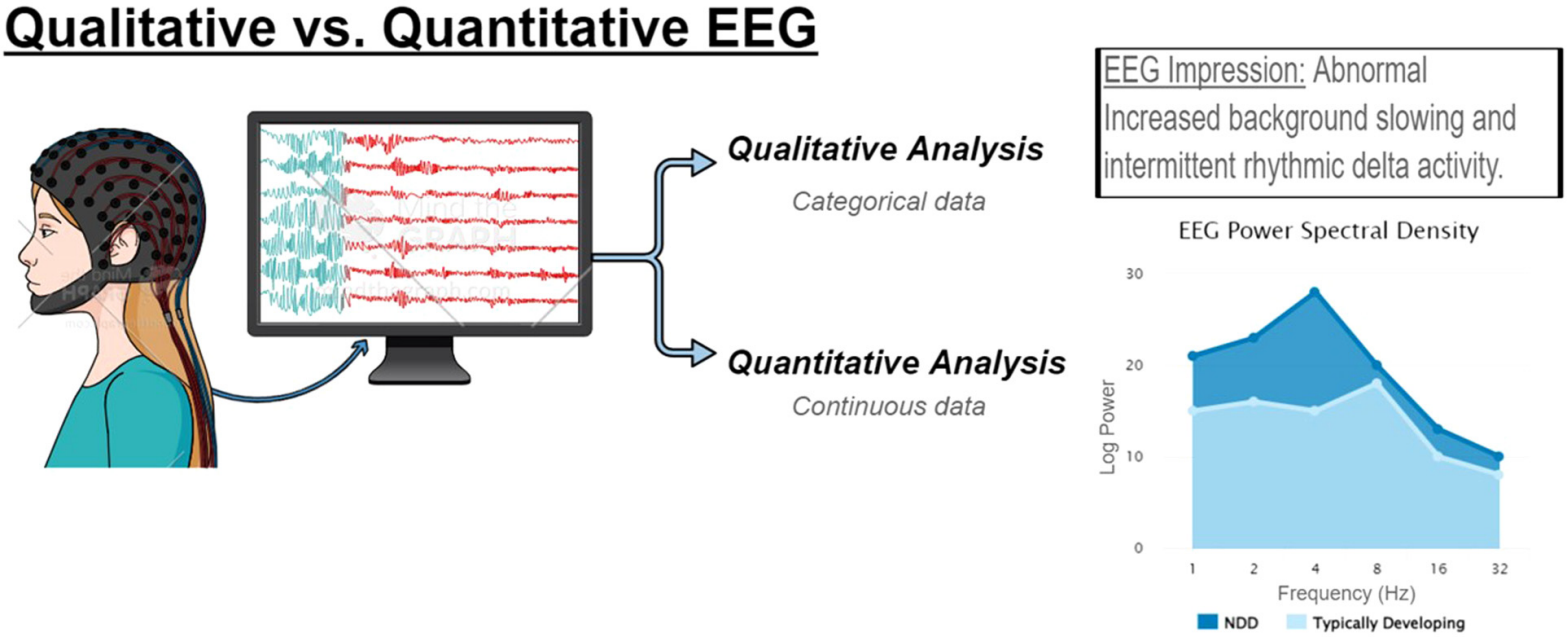
Electroencephalography (EEG) is a powerful tool to evaluate brain state and connectivity. As a clinical tool, EEG is used to evaluate for seizures, epileptiform discharges, and states of consciousness or encephalopathy. Qualitative review of EEG data generates a rich description of the record that can be captured as categorical data; however, quantitative analyses can also be applied to generated continuous data, such as power spectral density or measures of connectivity.

Scalp EEG dates back to the 1920s when Hans Berger, a German neuropsychiatrist, began recording scalp electrical potentials in patients with skull defects.^13^ In the 1930s, spike wave discharges that disrupted the brain's background oscillatory wave patterns were identified on EEG and linked to epileptic seizures.^14^ Decades later, visual inspection of scalp EEG remains the primary tool for evaluating, diagnosing, and managing patients with seizure disorders because it is a widely available and relatively inexpensive, noninvasive measure of brain function.^15^ The field advanced over the last century to electronic data recording and storage that has allowed for real-time EEG reading and increasingly complex quantitative analyses of the relationship between various frequency bands (eg, alpha, beta, gamma, theta, and delta) connectivity across brain regions that can detect quantitative differences that are not appreciated by conventional visual inspection (Figure 1).^16,17^ One of the most frequently used methodologies uses fast Fourier transform to quantify power at individual frequency bands averaged across the recording to build a power spectrum.^17^ From there, the relative power of each frequency band can be assessed. Coherence analyses can measure for synchronization between 2 channels or symmetry analyses can assess relative power between a symmetrical pair of electrodes to quantify regional brain connectivity. Today, EEG is being incorporated as a potentially diagnostic or translatable biomarker in clinical translational studies in many disorders, including genetic neurodevelopmental disorders and idiopathic autism spectrum disorder (ASD).^10,12,18^ Beyond its use in neurodevelopmental disorders and epilepsy, quantitative EEG analyses have been applied to Alzheimer's disease where changes in spectral power and phase coherence are emerging as promising clinical tools to assess severity of cognitive decline.^19^

In this review, we explore the use of EEG in 3 of the most prevalent genetic neurodevelopmental disorders—Angelman syndrome, Rett syndrome, and fragile X syndrome. These are 3 monogenetic disorders that are associated with developmental delay, epilepsy, and autism spectrum disorder that are at the forefront of drug development for neurodevelopmental disorders. Despite extensive observational and interventional studies in these conditions, there were no US Food and Drug Administration (FDA)–approved treatment for these disorders until 2023 when trofinetide was approved for Rett syndrome. With continued interest in development of robust biomarkers of neurodevelopmental disorders to measure target engagement or improvement in a clinical trial, we present a review of EEG biomarkers in 3 of the most well-known and well-studied genetic neurodevelopmental disorders.

## Methods

We searched Embase, MEDLINE, and MEDLINE EPub and InProcess databases using the following search terms: EEG and Angelman, EEG and Rett, and EEG and fragile X. Titles and abstracts were reviewed and only studies evaluating resting-state EEG in human participants published from 2012 to 2022 were included. EEG acquisition included standard clinical methods (10-20 EEG montage, 19 channels),^20–26^ 8-electrode montage,^27,28^ 128-channel dense array,^29–33^ and 26-channel electrode cap.^34,35^ Single case reports, studies of isolated event-related potentials (eg, auditory or visual evoked responses), and task-oriented EEG (eg, audiovisual task and working memory task) were excluded.

## Angelman Syndrome

Angelman syndrome (AS) is a neurodevelopmental disorder characterized by global developmental delay with moderate to severe cognitive impairment, limited expressive speech, impaired balance or ataxia, and characteristic patterns of behavior such as frequent laughter or hyperactivity.^36,37^ Angelman syndrome affects an estimated 1/12 000 individuals and is caused by maternally inherited loss of function variants or deletions involving *UBE3A*, paternal uniparental disomy of chromosome 15, or an imprinting defect.^38,39^ *UBE3A* encodes for a ubiquitin ligase and one consequence of loss of this protein inhibition of synaptic formation and remodeling.^40^ Children often have an ataxic gait and intractable epilepsy that can persist into adulthood, and prolonged seizures are associated with developmental regression.^36,41–43^ The characteristic EEG pattern of high-amplitude rhythmic slow-wave activity has long been recognized as a feature of Angelman syndrome and incorporated into diagnostic paradigms.^38,44–48^ Notched rhythmic delta had a specificity of 38% for Angelman syndrome and was identified as early as 14 months of age in a retrospective review of EEGs with the notched delta pattern or clinical Angelman syndrome phenotype.^49^ EEG is a powerful tool in the identification of potential patients with Angelman syndrome, and even a routine EEG (eg, 1-hour recording) can yield data on interictal discharges comparable to an overnight EEG if non-REM sleep, an activator of interictal discharges, is captured.^50^

A large prospective study of Angelman syndrome EEGs with blinded qualitative review revealed that more than 80% had intermittent rhythmic delta activity, followed by nearly 75% with interictal epileptiform discharges, and approximately 40% with intermittent rhythmic theta activity and posterior background slowing.^51^ On regression analysis, rhythmic delta activity decreased with age, but there was no clear correlation between EEG patterns and genotypes.^51^ In this study, the genotype classification for Angelman syndrome was used: (1) deletion class 1 (common 5.9-Mb deletion); (2) deletion class 2 (common 5.0 Mb deletion), atypical deletion (breakpoints and deletion sizes other than 5.9 or 5.0 Mb); (4) uniparental disomy (UPD); (5) UBE3A mutation (sequence variant); and (6) imprinting defects.^51^ Subsequent studies showed similar findings with a lack of correlation between genotype and EEG patterns. There was, however, an evolution of EEG patterns with age (eg, epileptiform activity shifting from posterior to anterior head regions with age) and increased delta activity during periods of uncontrolled seizures.^52,53^ It was not until later when quantitative analyses were applied to Angelman syndrome EEGs did correlations between delta activity, genotypes, and clinical severity emerge (see Table 1).

**Table 1.** Review of Quantitative EEG Analyses in Angelman Syndrome.^a^

Authors	Comparison Group (n)	Method	Results
Sidorov et al^33^	AS (26) HC^b^ (54)	Resting EEG • EEG power • EEG dynamics	Increased delta power Increased delta dynamics Delta power reduced with age, but did not normalize
Den Bakker et al^34^	AS (28), NREM sleep (13), Wake (26) HC^b^ (72), NREM sleep (54), wake (54)	Resting EEG • EEG power • Coherence map • Spindle detection	No difference in short-range coherence Increased long-range coherence in sleep and wake Increased delta power Decreased number and duration of sleep spindles
Frohlich et al^35^	Deletion AS (37) Nondeletion AS (30) HC (48)	Resting EEG • EEG power • Peak frequency	Increased delta power (deletion > nondeletion) Decreased beta power (deletion < nondeletion)
Frohlich et al^38^	AS (35)	Resting EEG • EEG power • Signal complexity (modified multiscale entropy)	Increased signal complexity in wake vs sleep EEG when controlling for delta power
Hipp et al^36^	AS (45)	Resting EEG • EEG power	Increased delta power correlated to increased clinical severity and earlier onset epilepsy
Ostrowski et al^37^	AS (82)	Resting EEG • EEG power	Inverse relationship between delta power and cognition, receptive and expressive communication, and fine motor skills

Abbreviations: AS, Angelman syndrome; EEG, electroencephalography; HC, healthy control; NREM, non–rapid eye movement.

^a^ Across all studies, there was increased delta power, and relative delta power tended to correlate to increased phenotypic severity.

^b^ Age-matched.

Quantitative analysis of EEGs from patients with Angelman syndrome and a mouse model of Angelman syndrome revealed that rhythmic delta activity was a reliable EEG biomarker in Angelman syndrome.^54^ In this study, delta power was significantly increased in comparison to age-matched healthy controls in both human and mouse model EEG.^54^ Delta dynamics (eg, degree of variability in the strength and duration of delta activity) were similarly increased in both mouse and human EEGs.^54^ In human EEGs, sleep and wake EEG epochs were reviewed independently and both showed increased delta power and dynamics compared with healthy controls.^54^ Subsequent studies reaffirmed the increased delta power in Angelman syndrome EEGs and found abnormal patterns of connectivity and irregularities in sleep, including decreased number and duration of sleep spindles.^55^ Further, these quantifiable EEG patterns correlated to genotype and symptom severity. When controlling for age, delta power was higher and beta power was lower in patients with deletions compared with nondeletion Angelman syndrome.^56^ Delta power was stable over time in those with longitudinal EEGs and tended to decrease with age at a similar rate as typically developing controls.^56^ When controlling for age, stronger delta power also correlated with earlier onset of epilepsy and lower performance across many developmental domains and adaptive abilities.^57^ Delta power was also predictive of cognitive abilities in Angelman syndrome after controlling for genotype and age.^58^ Because hypersynchronous slow wave activity is typically associated with unconscious states (eg, sleep or coma), investigators assessed signal complexity in awake vs sleep EEG in Angelman syndrome and found that wakeful EEG demonstrated increased signal complexity (eg, increased signal entropy) when controlling for delta power, suggesting that although hypersynchronous slow wave activity is commonly associated with unconscious states, it is not specific.^59^

With extensive EEG biomarker development studies across species, ages, and genotypes, EEG is beginning to be incorporated into clinical trials to assess the efficacy of novel therapeutics.^60,61^ In a post hoc analysis of the efficacy of minocycline in Angelman syndrome, there was a progressive reduction in global delta power and spike frequency posttreatment that paralleled the delayed improvement in other clinical outcome measures included in the study (eg, communication and fine motor ability).^60^ In preclinical studies of the efficacy of a lentivector-based stem cell gene therapy, the clinical phenotype (eg, motor, cognition, behavior) was rescued and delta power normalized in both newborn and adult mouse models of Angelman syndrome.^62^ And, in a randomized, double-blind, placebo-controlled study of exogenous ketones in Angelman syndrome, there was a significant improvement in fine motor skills and a reduction in delta power in the ketone formulation treatment arm.^63^ It is likely that increased delta power is a marker of neuronal dysfunction and disrupted connectivity rather than being specific to Angelman syndrome because increased delta activity can be seen in other disorders, including Rett syndrome and disorders of consciousness without a genetic linkage.^59,64^ If delta power is a global marker of neuronal dysfunction, perhaps that explains why relative delta power correlates with changes in severity of a broad range of clinical symptoms.

## Rett Syndrome

Rett syndrome is an X-linked neurodevelopmental disorder that predominantly affects girls. It was initially described by Dr Andreas Rett in the 1960s in girls who met their early developmental milestones followed by progressive neurologic decline and epilepsy, but the precise clinical definition of Rett syndrome has evolved over time (reviewed by Dolce et al).^65,66^ Multiple genes have been associated with the Rett syndrome phenotype, but in this review we limit discussion to the EEG characteristics of Rett syndrome associated with variants in *MECP2*, methyl-CpG-binding protein 2 found on chromosome Xq28.^67,68^ *MECP2* impacts multiple cellular mechanisms including modulating transcription of DNA and synaptic activity.^69^ The clinical progression of Rett syndrome has been well described and divided into 4 stages (see Table 2).^65^ In general, girls have relatively normal development in infancy with onset of regression after 6 months of age and deceleration of head growth. More than half of patients with Rett syndrome will develop epilepsy, and progressive motor and cognitive decline throughout childhood is common.^65^ Similar to Angelman syndrome, characteristic EEG patterns have emerged in Rett syndrome, although they are not necessarily specific to Rett syndrome.

**Table 2.** Clinical and Electrophysiological Stages of Rett Syndrome Natural Progression.^a^

Rett syndrome stage	Age	Clinical characteristics	EEG findings
1. Early onset stagnation stage	6 mo to 2 y	Developmental arrest Changed communication and personality Diminished play interest Hand washing stereotypy Decelerating head growth	Normal If abnormal: mild background slowing, spike wave discharge in sleep
2. Rapid destructive stage	2 to 4.5 y	Developmental regression with autistic traits and dementia Apraxic/ataxic gait Loss of hand skills Onset of seizures and hyperventilation	Normal to mild background slowing Centrotemporal spike or sharp waves Poorly formed sleep (loss of non-REM) Atypical spike and wave discharges enhanced by sleep
3. Pseudostationary/plateau stage	2 y to adulthoodSome patients remain in stage 3 for their entire lives	Increased attentiveness and decreased autistic traits Intellectual disability Gross motor dysfunction and apraxia Seizures	Poorly formed posterior dominant rhythm Low-voltage, poorly reactive, generalized slowing Poorly formed sleep architecture (absent non-REM) High-voltage delta and spike wave in sleep Multifocal spike wave and rhythmic bursts of central delta activity
4. Late motor deterioration stage	>10 y	Decrease or loss of mobility Decreasing seizures Growth retardation and weight loss	Marked background slowing with fronto-central rhythmic delta Multifocal spike wave Generalized slow spike wave in sleep Epileptiform activity may be absent

Abbreviations: EEG, electroencephalography; REM, rapid eye movement.

^a^ Rett syndrome progresses from stage 1 where patients have a stagnation of developmental gains and normal to mildly slow background on EEG to a period of rapid developmental regression with increasingly abnormal EEG findings, and finally a plateau phase with stable developmental disability and signs of encephalopathy on EEG. Some patients progress to further motor deterioration and decreasing seizure burden with reduced epileptiform activity on EEG.

Studies of characteristic EEG patterns in Rett syndrome date back to the 1980s. Early studies demonstrated that rhythmic slowing was the most common abnormality detected on EEG in both disorders, followed by spike-wave complexes.^44,70,71^ In Rett syndrome, EEG progressed to ill-defined low-voltage recordings with age.^70,72^ This EEG deterioration in Rett syndrome was further categorized into stages from normal (stage 1) to poorly formed low voltage background (stage 4) (see Table 2).^65,73^ Rhythmic theta in the central region has also been identified as a notable feature on EEG in patients with Rett syndrome and hypothesized to be indicative of impaired motor control due to frontal lobe dysfunction.^74^ Because of these patterns, EEG has been used as a powerful diagnostic tool in differentiating children with Rett and Angelman syndrome even before genetic testing was clinically available.^75^ Early studies investigating genotype-phenotype correlations did not identify any difference between the EEG pattern of patients with truncating vs missense variants of *MECP2.*^76^ Despite a lack of clear correlation with genotype, clinical EEG abnormalities do correlate with clinical severity and progression of disease, findings that have led to studies evaluating the use of EEG as a translational biomarker of disease severity, though characteristic findings have varied among the limited number of studies available (see Table 3).^77,78^

**Table 3.** Review of Quantitative EEG Analyses in Rett Syndrome.^a^

Authors	Comparison group (n)	Method	Results
Ammmanuel et al^43^	RTT with overnight EEG (10) HC^b^ with PSG (15)	Sleep EEG • EEG power • Sleep stages	Increased delta power Decreased slow-wave sleep cycles Negative correlation with seizures
Keogh et al^59^	RTT-*MECP2* (36) RTT-*CDKL5* (4) Longitudinal collection (9) *MECP2* (7), *CDKL5* (2)	Resting EEG • EEG power • Hemisphere activity asymmetry • Connectivity	Dysfunctional network architecture correlates to phenotypic and genotypic differences Spectral power decreases with age, but network coherence remained stable
Roche et al^60^	RTT (57) HC (37)	Resting EEG • EEG power	Decreased power in middle frequency bands Increased delta and theta power in the postregression phase

Abbreviations: EEG, electroencephalography; HC, healthy control; PSG, polysomnography; RTT, Rett syndrome.

^a^ Studies demonstrate increased power of slow-wave frequencies (eg, delta and theta), reduced power in middle-frequency bands, and abnormal network connectivity.

^b^ Age-matched.

Similar to findings in Angelman syndrome, a study of overnight EEG in girls with Rett syndrome discovered a significantly higher delta power in slow-wave sleep in comparison to age-matched girls without Rett syndrome despite a reduction in the number of slow-wave sleep cycles.^64^ The abnormalities were driven by the younger age group and negatively correlated to the incidence of seizures (eg, lower number of slow-wave sleep cycles in those with seizures).^64^ This was one of the first studies in Rett syndrome to translate an EEG finding (eg, sleep cycle dysfunction) from discoveries in *Mecp2*-deficient mice to humans and suggested that quantitative EEG analyses could be incorporated into clinical trials.^64,79^ Subsequent studies have explored relationships between EEG abnormalities and phenotypic or genotypic differences. Keogh et al^80^ explored the relationship between EEG abnormalities and phenotype by dividing cases into the classic phenotype (most common), Hanefeld variant (rare severe), and preserved speech variant (PSV, rare mild) and accounted for severity of epilepsy (eg, no epilepsy, epilepsy, or treatment-resistant epilepsy). In measures of network connectivity, Rett syndrome–*MECP2* participants demonstrated a pattern of low covariance in left occipital-temporal pairs and reduced involvement of right occipital-temporal pairs.^80^ When comparing variable phenotypes (eg, classic vs Hanefeld vs preserved speech variant), there was no significant difference in EEG power or hemispheric variance, though there was a trend toward higher power in high-range frequencies in classic Rett syndrome and left hemisphere asymmetry in Hanefeld and preserved speech variant (PSV) Rett syndrome.^80^ There was a significant difference in network connectivity between the phenotypes where the classic and preserved speech variant groups had an overall pattern of low network activity in bioccipital regions and the Hanefeld group had low covariance in the delta band.^80^ Finally, on longitudinal analysis 10-14 months after the initial EEG, there was a decrease in spectral power that was most notable in the left frontal and parietal regions.^80^ When comparing severity of epilepsy, there was no difference in overall spectral power or among individual frequency bands between the 3 groups, but there was a pattern of increasing left occipital predominance with increasing severity of epilepsy and differences in overall network architecture.^80^ Finally, Roche et al^81^ demonstrated an increased power in the low-frequency bands (eg, delta and theta) in the postregression phase that correlated to lower scores on standardized cognitive assessment measures.

EEG has been used as a clinical efficacy outcome measure in clinical trials of Rett syndrome. In a study of Cerebrolysin (cerebroprotein hydrolysate), treatment with this brain-derived peptidergic drug improved behavioral activity, attention, and motor function in addition to reducing theta activity, increasing beta activity, and restoring occipital alpha rhythms.^82^ In another study of recombinant human insulin-like growth factor 1 (IGF1) in girls with pathogenic variants in *MECP2*, improvements in anxiety and depression combined with reversal of the right frontal alpha band asymmetry on EEG was seen after treatment.^83^ However, subsequent studies failed to demonstrate a significant change in this EEG pattern, and another study with dextromethorphan demonstrated behavioral rescue without any effect on epileptiform discharges on EEG.^84–86^ Glatiramer acetate has also demonstrated reduction in EEG abnormalities (eg, decreased epileptiform discharges).^87^ Finally, Keogh et al^88^ investigated the impact of IGF1 on network formation in responders in comparison to nonresponders based on clinical assessments. There was a significant difference in network measures that was predicted with 100% accuracy using a machine learning model applied to pretreatment data and highlighted the promise that quantitative EEG analysis has a role as a biomarker in clinical trials.^88^ Rett syndrome may also be a candidate for gene replacement therapy as even postnatal rescue of the genotype demonstrates rescue of the behavioral, motor, and EEG phenotype.^89^

## Fragile X Syndrome

Trinucleotide repeat expansions result in silencing of expression of the *FMR1* gene on the X chromosome causing fragile X syndrome (FXS), the most prevalent monogenetic causes of global developmental delay and autism spectrum disorder accounting for approximately 1% of cases.^9^ Decreased or absent fragile X messenger ribonucleoprotein (FMRP), the protein product of *FMR1*, leads to abnormal dendritic pruning, an increased number of dendrites in addition to increased dendritic length, and abnormal synaptic plasticity.^90^ Most patients with fragile X syndrome (full mutation with >200 CGG repeats) are male, but females with fragile X syndrome may also have developmental disability.^90^ Many clinical trials of treatments that target the underlying pathology of fragile X syndrome have been evaluated in clinical trial, but none have demonstrated sufficient benefit to be approved.^9^ Further, fragile X syndrome is a candidate disorder for viral-mediated gene transfer therapy and preclinical studies demonstrated rescue of social deficits and abnormal slow-wave activity on EEG in male *Fmr1*-knockout rats treated with AAV9-*FMR1.*^8,91^ EEG is emerging as a potential biomarker to be incorporated into future clinical trials (see Table 4).

**Table 4.** Review of quantitative EEG analyses in fragile X syndrome.^a^

Authors	Comparison group (n)	Method	Results
Van der Molen et al^73^	FXS (8 M) HC^b^ (12 M)	Resting state • EEG power	Increased theta Decreased upper alpha
Van der Molen et al^74^	FXS (8 M) HC^b^ (12 M)	Resting state • EEG power • Connectivity	Reduced alpha and beta connectivity Increased theta connectivity Increased path length in theta bands
Wang et al^75^	FXS (15 M, 6 F) HC^b^ (15 M, 6 F)	Resting state • EEG power • Connectivity • Cross-frequency amplitude coupling	Increased gamma Increased theta-gamma coupling Decreased alpha-gamma coupling
Proteau-Lemieux et al^76^	FXS (16 M, 10 F) HC (40 M, 37 F)	Resting state • EEG power • Alpha peak frequency • Multiscale entropy (MSE)	Increased gamma Decreased alpha Decreased alpha peak frequency No difference in theta/beta ratio Decreased signal complexity using MSE
Smith et al^78^	FXS (28 M, 23 F) HC^b^ (29 M, 19 F)	Resting state • EEG power • Alpha peak frequency	Increased theta (M/F) Increased low alpha (F) Decreased beta (F) Decreased gamma (F) Decreased epsilon (F)
Wilkinson et al^77^	FXS (11 M) HC^b^ (12 M) Cognitive-Matched HC (12 M)	Resting state • EEG power	Increased gamma

Abbreviations: F, female; FXS, fragile X syndrome; HC, healthy control; M, male.

^a^ Studies show patterns of increased spectral power in the gamma frequency band, reduced power in the alpha frequency band, and abnormalities in network connectivity.

^b^Age-matched.

Among individuals with FXS, retrospective studies of EEGs obtained for a clinical suspicion of seizures demonstrated background slowing and epileptiform discharges.^92,93^ Of the 8 individuals with repeat EEG, posterior dominant rhythm normalized in 5, whereas 2 patients had stable abnormalities and 1 demonstrated worsening.^93^ In patients with fragile X syndrome, resting state EEG with eyes closed has shown increased theta power and reduced alpha power, suggestive of an imbalance of excitatory and inhibitory cortical circuit activity.^35^ In further analyses of brain connectivity, van der Molen et al^34^ applied graph theory to assess the neural architecture of anatomical and functional connectivity across brain regions within spectral bands and demonstrated findings suggestive of immature cortical networks. In typical development, synaptic growth and pruning are associated with decreasing delta and theta power coupled with increasing long-range alpha and beta density. In individuals with fragile X syndrome, the opposite was seen with a decrease in functional connectivity within alpha and beta frequency bands and increased connectivity within theta frequency bands.^34^ Further, there was increased path length in theta bands compared to healthy controls. Together these findings support the theory of impaired cortical maturation in fragile X syndrome.^34^

In a case-control study of resting dense-array EEG in 21 individuals with nonepileptic fragile X syndrome, findings suggested a net hyperexcitability with increased gamma and decreased alpha frequency band power that correlated with impaired social skills (eg, measured by the Social Communication Questionnaire) and sensory processing dysfunction (eg, measured by the Sensory Profile).^94^ Further, there was a U-shaped pattern of relative EEG power with increased power in the theta and gamma frequency bands and reduced power in the alpha bands, which was similar to findings previously reported in idiopathic autism spectrum disorder.^94^ There was also an increase in theta-gamma coupling and increased connectivity in gamma band activity, along with reduced alpha power and connectivity.^94^ Together these findings suggested that circuits in the fragile X syndrome brain rely on the less effective top-down inhibitory control of the theta frequency band to downregulate high-frequency (eg, gamma) activity, rather than the more typical modulation by alpha oscillations, which may correlate to cognitive impairment and behavioral rigidity seen in fragile X syndrome.^94^

Building upon these studies, Proteau-Lemieux et al^30^ explored signal complexity on fragile X syndrome EEGs and again demonstrated increased gamma power, decreased alpha power as well as reduced alpha peak frequency. The finding of increased theta/beta power commonly seen in patients with attention-deficit hyperactivity disorder (ADHD) was not seen in fragile X syndrome, but they did find an increase in delta power.^30^ Signal complexity, which typically increases with age and is a sign of cortical maturation, was reduced in the central and temporal regions using multiscale entropy in individuals with fragile X syndrome and may correlate to the growing discrepancy between chronological age and developmental abilities seen in fragile X syndrome.^30^ Surprisingly, in a study assessing the relationship power spectrum and language abilities in young boys with fragile X syndrome, there was a positive correlation between gamma power and better language abilities.^33^

Across all studies, individuals with fragile X syndrome demonstrate increased gamma power and decreased alpha power in comparison to healthy controls, suggestive of cortical hyperexcitability and impaired cortical maturation.^30,34,94^ FXS mostly affects males, but even when accounting for gender in the above studies, there was minimal to no difference between affected males and females.^30,94^ However, in one study assessing the sex differences in resting EEG power, males and females with fragile X syndrome both had increased theta power, but females had an increase in low alpha and a decrease in beta, gamma, and epsilon power.^31^ With the promise of reproducible findings across sites and the age spectrum of fragile X syndrome, EEG may become an important tool to combine with proxy-report clinical outcome measures in clinical trials of fragile X syndrome, but recent studies have incorporated event-related potentials rather than resting state EEG.^91,95^

## Discussion

The field of rare neurodevelopmental disorders faces many challenges including overlapping phenotypes between disorders as well as variable clinical presentations and severity within single-gene disorders. Further, the available assessment tools are limited in their ability to measure and discriminate between clinical phenotypes accurately and reliably. There is also a lack of approved targeted treatments, despite promising results of novel therapeutics in preclinical studies. The failure of novel treatments to achieve success in clinical trials is in part due to a lack of objective biomarkers of disease severity in addition to lack of clinical outcome measures capable of capturing symptom improvement within the time frame of a clinical trial. The challenges of accurate and reliable phenotypic characterization impact biomarker development, especially when the biomarker correlates with many aspects of the disorder, as is the case with delta power in Angelman syndrome. An established biomarker can be a powerful tool that can aid in patient selection as well as serve as an outcome measure to assess response to intervention. A neurophysiological biomarker should have adequate test-retest reliability, be suitable to use as a repeated measure, demonstrate a consistent relationship to disease phenotypes, and be scalable across a variety of clinical settings.^96^ EEG is a noninvasive measure of brain electrical activity that is readily available in most major medical centers that can be repeated more frequently than most psychometric tests. However, quantitative analytic capabilities may be more limited to specialty centers and require additional training and expertise to execute accurately. The EEG epochs analyzed must be selected carefully including the physiological state of the patient, absence of artifact, and consideration of concomitant medications. Despite the current limitations, commercial EEG software is evolving to include capabilities to generate quantitative analyses, including power spectral analysis, and it is possible that it will become more widely available in coming years. Another advantage of EEG is that it is less susceptible to bias as the EEG assessor may be blinded to participant characteristics or treatment allocation and does not rely on the recall or assumptions of the proxy-reporter (eg, the parent or caregiver). Finally, EEG is a translatable assessment tool that can be applied to both animal models of disease and human patients. Many preclinical studies rely on behavioral assays of learning and memory or social behaviors that are loosely correlated to clinical symptoms in human patients. Adding an objective measure like EEG provides an extra layer of confidence in the efficacy read-outs in preclinical studies of novel interventions.

In this review, we discussed the findings from studies that incorporated analysis of EEG, with a focus on quantitative analysis, in 3 of the most common genetic neurodevelopmental disorders, Angelman syndrome, Rett syndrome, and fragile X syndrome. Phenotypically, all 3 disorders are characterized by global developmental delay with severe impairments in expressive speech and autism spectrum disorders. Although epilepsy is more prevalent in Angelman syndrome and Rett syndrome, a subset of individuals with fragile X syndrome will also have seizures. Importantly, however, the degree of developmental disability is not completely dependent on seizure control in any of these conditions. Despite variability in epilepsy severity, quantitative EEG analyses among these 3 disorders demonstrate patterns that have been reproduced across multiple sites. Early studies described the characteristic, qualitative abnormalities on EEG such as background slowing and epileptiform discharges. However, when using quantitative methods, such as power spectral analysis, background slowing may be calculated as relative delta power, as was the case in Angelman syndrome. This transforms the categorical variable of background slowing into a continuous variable, which holds more statistical power and provides the opportunity for increasingly complex statistical analyses. This does not discount the importance of the qualitative EEG review, which provides the clinician with valuable information that impacts medical decision making, but rather demonstrates how EEG may be a versatile tool with the ability to use variable analytical techniques depending on the question being asked.

Although the differences in power spectral density seen in these 3 disorders are not specific, the results were reproduced across multiple sites, in different participant populations, and using different data acquisition techniques. Although a detailed review of the animal model studies was beyond the scope of this article, all 3 disorders have demonstrated similar EEG findings in their respective models. Further, in the case of Angelman syndrome and Rett syndrome, EEG has been incorporated into clinical trials and demonstrated a response to intervention in some studies and post hoc analyses. Although early studies considered the diagnostic power of EEG, with the increased accessibility of genetic testing and trend toward defining developmental syndromes by a single gene, EEG may be an additional tool to aid in phenotypic characterization and tracking disease progression over time. However, as more individuals with rare genetic syndromes are identified, the spectrum of each disease is likely to continue to expand, as we have seen over time in the cases of these 3 exemplary disorders. It is plausible that EEG could be an objective tool used to differentiate between disease phenotypes as there is precedence for EEG correlation with genotype, cognitive abilities, and severity of seizure burden. This suggests there may be value in EEG assessments beyond the evaluation for seizures, although further studies are needed to investigate the clinical relevance of EEG abnormalities outside the realm of epilepsy management.

Future studies may continue to build on this foundation in both qualitative and quantitative analysis of resting-state EEG. Because resting-state EEG is readily available in most clinical centers, it has value in scalability, but likely limited in specificity. Even within our 3 disorders, Angelman syndrome and Rett syndrome both demonstrated increased delta power, whereas fragile X syndrome demonstrated increased gamma power. The underlying pathomechanisms of neuronal dysfunction that generate these differences in power spectral density on scalp EEG will need additional investigation. However, there may be future avenues of exploration in assessing measures of network connectivity in resting state, sleep, and task-oriented EEG paradigms that could yield increased disease-specificity or symptom-specificity.

## Conclusion

In conclusion, EEG is a noninvasive, objective biomarker of brain connectivity that can correlate to disease severity. EEG is currently used primarily to assess patients with seizurelike activity and management of epilepsy. However, beyond its applications in epilepsy, EEG is emerging as a potential biomarker of disease severity in Angelman syndrome, Rett syndrome, and fragile X syndrome. There are hundreds of genetic neurodevelopmental disorders that have promising targeted therapies in development but are lacking appropriate clinical outcome measures and objective biomarkers of disease. With the precedent set by Angelman syndrome, Rett syndrome, and fragile X syndrome, EEG could be explored as a candidate biomarker in many other neurodevelopmental disorders in the future.
